# Supplementary figures and images for: Trim66’s paternal deficiency causes intrauterine overgrowth
Source: Life Sci Alliance. 2024 May 7;7(7):e202302512. doi: 10.26508/lsa.202302512 (PMC11077763; doi:10.26508/lsa.202302512)

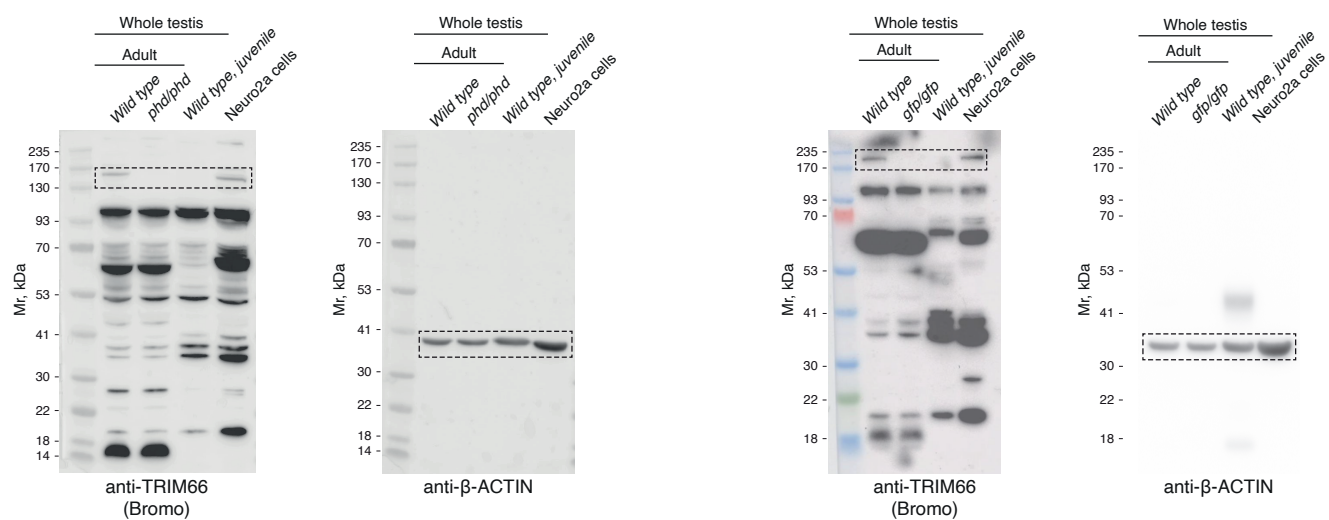

**Source data. Uncropped westernblots (Figs 2B,C).**

Supplement: Supplementary file 1 [file LSA-2023-02512_SdataF2.pdf]
